# Where Reality and Fantasy Collide—Prolonged Fever to Munchausen Syndrome by Proxy

**DOI:** 10.3390/children11121482

**Published:** 2024-12-04

**Authors:** Raluca Maria Vlad, Ruxandra Dobritoiu, Alina Turenschi, Daniela Pacurar

**Affiliations:** 1 Department of Paediatrics, “Carol Davila” University of Medicine and Pharmacy, 050474 Bucharest, Romania; raluca.vlad@umfcd.ro (R.M.V.); alina.burcuta@drd.umfcd.ro (A.T.); daniela.pacurar@umfcd.ro (D.P.); 2 “Grigore Alexandrescu” Emergency Children’s Hospital, 011743 Bucharest, Romania; 3 Ploiesti Children’s Hospital, 100336 Ploiești, Romania

**Keywords:** prolonged fever, painful joints, hemoptysis, hematuria, Munchausen syndrome

## Abstract

**Background:** Munchausen syndrome by proxy (MSBP) or factitious disorder imposed on another (FDIA) is a bizarre psychiatric entity, consisting of the fabrication of symptoms and alteration of laboratory tests by a caregiver. It is considered a serious form of child abuse. Alarm signs are frequent medical visits and strange symptoms that are never objectified during hospitalization. **Methods:** This case sets a bright light on how difficult the diagnosis and management of FDIA is and the severe consequences this disease has on a defenseless child. **Results (Case report):** A boy who is 3 years 8 months old first presented to our department in October 2022 for prolonged fever. We ruled out infections, malignancies, and autoimmune diseases. The patient kept coming back once every fortnight for the same reason—fever, every time associating it with something in particular and new—from painful joints to hemorrhagic complaints. Interestingly, with every new visit, the patient’s medical history became more complex. The mother also developed an attachment relationship with the medical staff. During a 4-month period of repeated admissions, the child’s symptoms were never objectified throughout hospitalization, and never consistent with the declared symptoms and test results. When the physician’s attitude changed from empathic to distant, she never came back for check-ups. **Conclusions:** A pediatrician’s work largely depends on good communication with the parents. When there is no medical explanation for declared symptoms, one might question the merit of the story.

## 1. Introduction

In 1951, Richard Asher described a bizarre entity in the form of child maltreatment called “Munchausen syndrome by proxi” (MSBP), consisting of the fabrication or induction of signs and symptoms, as well as the alteration of laboratory tests by a caregiver. Nowadays, this psychiatric pathology is recognized as a factitious disorder imposed on another (FDIA) and is considered to be a very serious, potentially deadly, form of child abuse. Alarm signs are frequent medical visits in numerous clinics and with numerous doctors, and strange symptoms that are never objectified during hospitalization [1,2,3,4,5].

Even though it has been previously described as a rare disorder, these days, cases are frequently encountered, one stranger than another, with serious and life-threatening presenting symptoms of the abused child [4]. The mortality rate of children maltreated by MSBP adults reaches 10% [6]. The true prevalence of MHBP syndrome remains unknown, probably because many cases are overlooked [1]. Less severe cases are not reported and some may remain undetected because of the hidden nature of the abuse. Maltreatment usually begins early in a child’s life, with the average age at diagnosis of children exposed between 20 and 40 months [4,7].

The perpetrators of MSBP are usually mothers, but cases where fathers were maltreating their children have been cited in the literature [1,5,6]. Caregivers are usually well-educated persons, some of them actually having a medical background. This allows them to foresee physician’s questions or investigation proposals, making the diagnosis process tricky [1,7].

The DSM-5 (Diagnostic and Statistical Manual of Mental Disorders) defines MSBP with the following criteria, which must always be met: (1) the perpetrator engages in the deceptive falsification of physical or psychological signs or symptoms, or of induction of injury or disease in another; (2) the perpetrator presents the victim to others as ill, impaired, or injured; (3) the deceptive behavior is present also in absence of external incentives; and (4) the behavior is not better accounted for by another mental disorder (psychotic or delusional disorder) [2,3,6,8].

**Figure 1:**
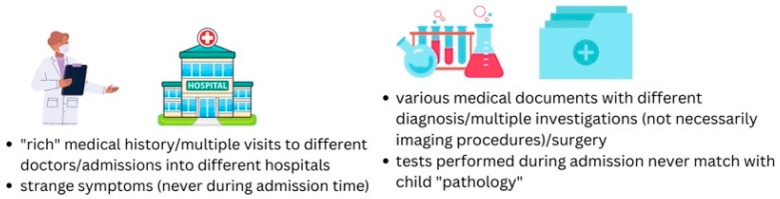
Alarming signs of MSBP.

It is of crucial importance that a physician, having even the slightest suspicion, gathers signs of this disorder. A child with recurrent symptoms that have never been objectified during hospitalization and never matched by expected laboratory results should raise a red flag, as well as the worsening of these symptoms every time the discharge moment approaches. A treatment course that is not clinically consistent, or a mother who seems too calm despite the child’s serious illness and who welcomes even painful medical examinations for her child should trigger suspicion among the medical staff (Figure 1). There are more indicators of this psychiatric disorder, such as peculiar family medical background (sudden unexplained deaths), the father being emotionally distant or absent from the child’s life, and the changing of declared residence with every new admission [2,3,4,5,6,7].

However, it is important to distinguish MHBP syndrome from other similar entities, such as the anxiety of the caregiver, who simply has excessive care for the child, but is not abusive. Poor compliance with treatment is another issue, resulting in the child’s persisting or worsening of illness. Also, malingering the child with an external purpose (obtaining financial benefits, for example) should be ruled out [2,3,6].

**Figure 2:**
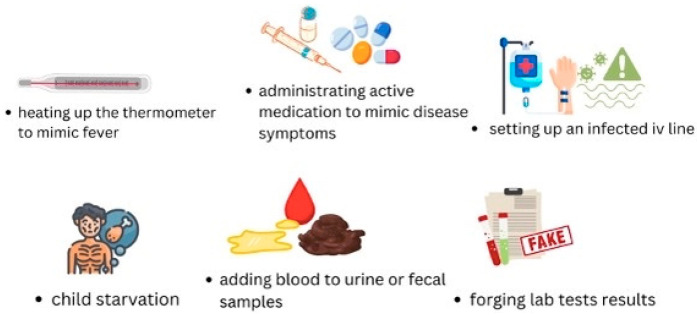
Severe symptoms of caregivers with MHBP syndrome.

Among the commonest signs and symptoms described in children with MSBP are seizures, unexplained metabolic and hydroelectrolytic disturbances, unexplained bleedings, fever with no explanation, recurrent pain in various sites, genital or skin injuries, subcutaneous emphysema, or “accidental” poisoning. All conditions have one thing in common—they are easily inducible by another person. This brings us to the most severe behavior of caregivers with MSBP, as they might be capable of anything [9,10,11,12,13,14,15,16,17,18] (Figure 2).

## 2. Case Series

A boy who is 3 years 8 months old first presented to our department in October 2022 for a continuous fever for 21 days (daily, with pause periods no longer than 12 h) and pain in lower limbs for 3 weeks. He had previously received at-home antipyretic medication and oral antibiotic treatment, without fever remission. Initially, he did not associate cutaneous rash, pain or joint swelling, abnormal stools, or reno-urinary symptoms, but his mother described episodes of nocturnal sweets, which started roughly at the same time as the fever.

From the patient’s family history, it is important to mention that his mother had acute lymphoblastic leukemia at age 14 and a chest tumor surgically removed at age 18. His father was in good health. The patient’s medical history did not reveal anything significant.

Clinical examination at admission identified no fever, normal growth, normal parameters of cardiovascular, respiratory, and digestive functions, muscle pain in lower limbs, malformed ears, and left testicular agenesis.

The patient underwent complex investigations to exclude different causes of prolonged fever. Infectious diseases were ruled out through peripheral bacterial cultures and blood cultures, which were all negative (but we have to keep in mind that the boy received multiple courses of antibiotics prior to admission). The following viral infections were excluded: Epstein–Barr (EBV), Cytomegalovirus (CMV), Human Immunodeficiency Virus (HIV), Hepatitis B Virus (HVB), and Hepatitis C Virus (HVC). IgG for SARS-CoV-2 came out positive, but there were no arguments for long-COVID-19 syndrome. Tuberculosis was excluded via negative QUANTIferon and normal radiologic imaging. Further, no inflammatory syndrome, normal values of immune blood testing, serum complementum, and immune circulating complexes made an autoimmune disease improbable. Abdominal and thoracic imaging ruled out solid tumors. Blood count with peripheral blood smear without anomalies and no atypical cells detected on medullary biopsy excluded hematological neoplasia.

**Figure 3:**
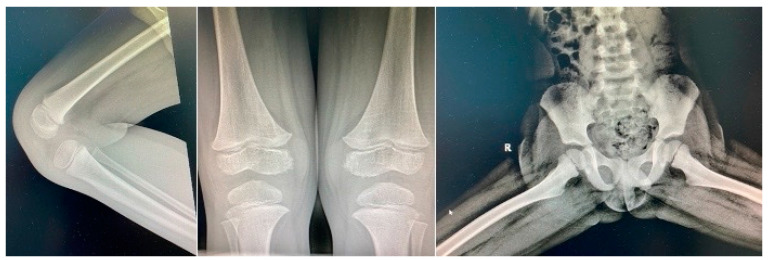
Normal bone X-rays.

Three weeks later, the patient came back with fever and left knee swelling with partial function impairment within 24 h of onset. His mother also described, two days prior, a non-itching maculo-papular cutaneous rash on the chest and belly, which disappeared spontaneously. Clinical examination did not encounter anything out of the ordinary (no fever, no rash), just mild painful swelling of the left knee. Laboratory investigations were also, this second time around, in the normal range. Borrelia infection was excluded. The orthopedic exam did not describe local inflammatory signs and joint mobility and knee X-rays were normal (Figure 3). He received oral anti-inflammatory medication.

The patient came back every two weeks for fever. Screening for solid tumors was performed and, again, the autoimmune pathology came back with normal results. Again, infectious diseases were excluded through negative central and peripheral bacterial cultures and viral serologies. Repeatedly negative QUANTIferon alongside normal imaging and specific pulmonology examination ruled out tuberculosis. Malignant causes were again excluded by normal blood count and medullary aspirate, as well as normal ultrasounds and X-rays. Idiopathic juvenile arthritis (IJA) remained a possibility, as the patient had prolonged fever, joint pain, and inflammatory syndrome with erythrocyte sedimentation rate higher than C reactive protein; he, however, did not meet the time criteria symptoms for at least 6 weeks. Anticitrullinated protein antibodies, anti-vimentin antibodies, and rheumatoid factor were negative; nevertheless, negative antibodies do not certify as exclusion criteria for IJA.

Two weeks later, the patient returned to our clinic with a fever and swallowed neck lymph nodes. Periodic familial fever syndrome (PFAPA) was also considered this time.

**Figure 4:**
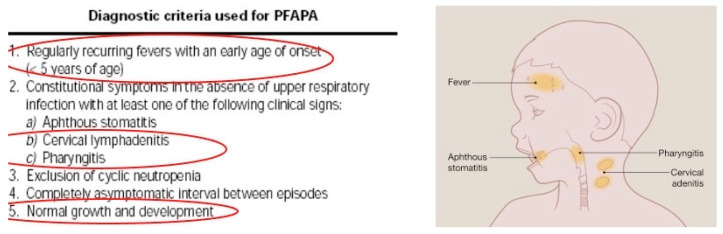
PFAPA criteria in our patient [19,20].

He was younger than 5 years, had recurrent fever, cervical lymphadenitis, pharyngitis, and normal growth and development (Figure 4). The therapeutic challenge was performed, a single dose of oral cortisone was administered with no response. The current lab analysis was again normal. The primary physician started to notice a pattern: declared symptoms (present at home), but never during hospitalization. Rare neoplasia was considered (cavum, brain). Magnetic Resonance Imaging (MRI) of the head and neck returned normal findings. The patient continued a course of prolonged, high fever. A combined course of antibiotic and antifungal treatments was started, with good results—the fever ceased temporarily.

Approximately one week after the previous hospital admission, the patient returned for fever and joint pain. But this time, the mother described a new symptom—hemoptysis. Also, the family medical history was now far more complex and abundant in various diseases: the father had long-time non-investigated cytolysis, the maternal grandmother had lung cancer, the maternal grand-grandfather had cerebral tumors, the paternal grandfather had prolonged nose bleeds with no certain cause, the maternal great-grandmother had gall-bladder cancer, and the first-line maternal cousin, aged 3, was diagnosed with nephroblastoma.

**Figure 5:**
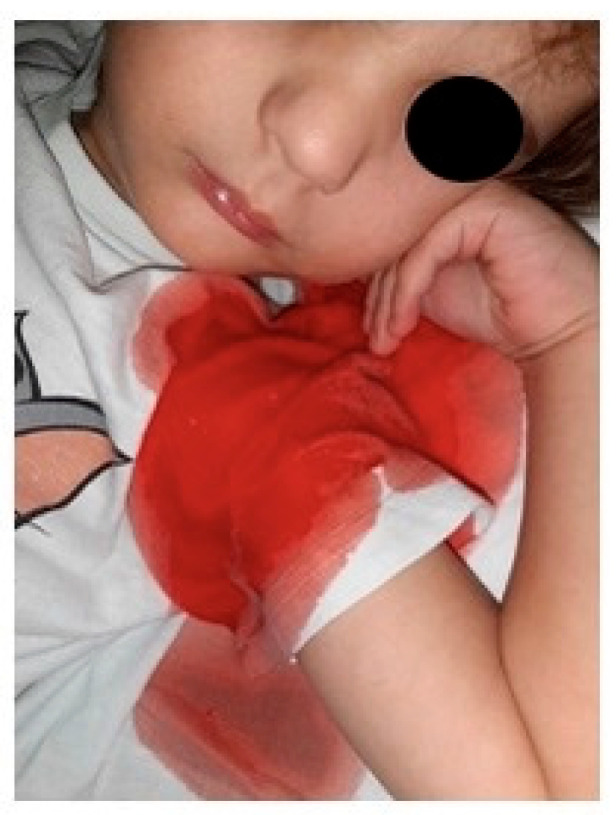
Proof of hemoptysis the mother repeatedly provided during admission.

This time around, tuberculosis was ruled out again (QUANTIferon, Bacillus Koch (BK) cultures, and polymerase chain reaction for BK were all negative). Congenital immunodeficiency was also taken into consideration. However, the boy did not experience recurrent, severe infections, in his first three years of life; immunogram, leukocyte immunophenotyping, and HIV had normal values. A full body scan computed tomography (CT) helped rule out nephroblastoma, neuroblastoma, and malignant head, chest, or abdominal tumors. Keeping in mind the so-called maternal line familial malignant memorabilia, genetic testing was recommended, and WES testing for hematological-linked syndromes and syndromes associated with multiple malignancies. The blood test was sent for evaluation, but, as we later discovered, the mother, although she had declared that she had filled in and sent the required consent forms, never did so. During this admission, the boy had numerous declared episodes of hemoptysis, the mother showed photographs on a daily basis, and no medical staff actually saw the bleeding episodes (Figure 5). Surprisingly, despite recurrent episodes of bleeding, the hemoglobin levels were always steady.

**Figure 6:**
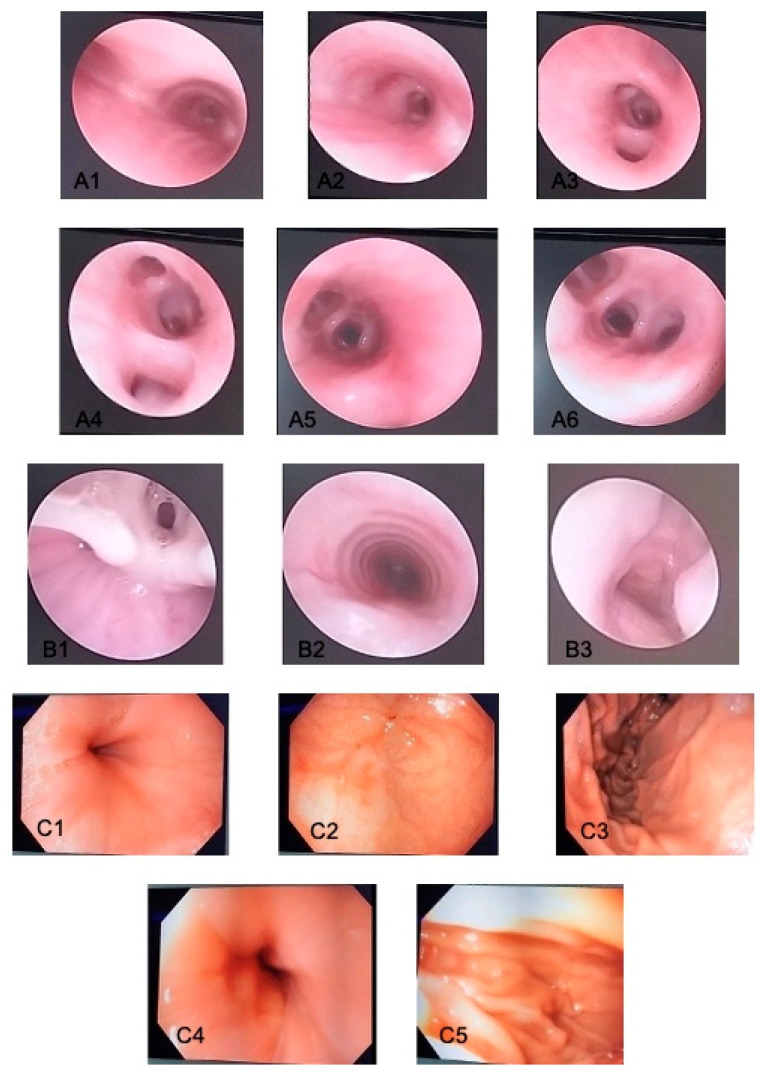
(**A**) Bronchoscopy: (**A1**) carina of trachea; (**A2**) right bronchus; (**A3**) bifurcation of right bronchus—no sign of bleeding, no malformations; (**A4**) terminal right bronchus; (**A5**) left bronchus; (**A6**) bifurcation of left bronchus—no sign of bleeding, no malformations. (**B**) Posterior nasal and cavum endoscopy: (**B1**) vocal cords; (**B2**,**B3**) cavum—no signs of active bleeding. (**C**) Upper digestive tract endoscopy: (**C1**) stomach’s cardia; (**C2**,**C3**) stomach—no active signs of bleeding; (**C4**) Pylorus; (**C5**) Duodenum—no signs of active bleeding.

A pan-endoscopy of the upper and lower respiratory tract was performed, with no signs of active or recent bleeding in the nose, cavum, bronchi (Figure 6A,B), and upper digestive tract (Figure 6C). Bronchial aspirate, gastric and bronchi lavage were negative for BK. Vascular MRI of the head, neck, and chest ruled out bleeding due to vascular malformation.

With complex hemostatic therapy, the hemoptysis episodes suddenly stopped. The patient was asymptomatic, with normal blood tests. On the day of discharge, a new symptom emerged—gross, painless, macroscopic hematuria with dysmorphic urinary erythrocytes. Complex coagulation testing returned normal results. The hemoglobin levels were still steady regardless of severe active bleeding. The patient had normal diuresis, no edema, hypertension, or nitrogen retention. Acute severe glomerulonephritis was now considered (although complete clinical criteria were not met) and the patient was started on intravenous cortisone and transferred to the Nephrology Department for further testing and treatment. Systemic vasculitis (Goodpasture syndrome or others) was now being considered (prolonged fever, recurrent hemoptysis, and hematuria). Anti-myeloperoxidase antibodies (pANCA), anti-nuclear antibodies (ANA), and anti-glomerular membrane antibodies were negative.

Over the 4-month course of repeated hospital admissions, the patient’s mother managed to develop an attachment relationship with the attending physician and the medical personnel. She was respectful and patient, and always calm and collected despite the serious illness of her child. She took advantage of the empathic attitude of her doctor and used details she learned from her physician’s private life to gain sympathy. She had an active social media presence, and took part in various profile groups, from where she constantly received validation and financial support. She constantly changed her declared home address from one admission to another. She declared that she was pregnant and having a difficult and complicated pregnancy, and presented herself as the sole caregiver of the child, the father being away on a military mission. At some point, she even declared having a medical background (2 years of medical school).

The medical personnel’s attitude in the Nephrology Clinic was different, perceived by the mother as “cold and distant”; as a result, in 5 days, the patient’s severe acute glomerulonephritis was inexplicably cured (from gross hematuria to a normal urine sample). A few hours after discharge from the Nephrology Clinic (normal clinical examination and blood tests on discharge), the mother returned to our clinic describing the sudden onset of abundant hematemesis. The hemoglobin level was normal. Upper gastrointestinal endoscopy was urgently performed, again, with no signs of active or recent bleeding. This was the turning point of the story, as the attending physician now put everything in a different perspective. During the 4-month period of repeated admissions, the child’s symptoms were never objectified during hospitalization, and never consistent with the declared symptoms and test results. Now suspecting Munchausen Syndrome by proxy, the doctor’s attitude changed suddenly from empathic to distant. The mother stopped receiving validation and sympathy, resulting in a subsequent paucity of complaints and the disappearance from follow-up.

## 3. Discussion

“To be or not to be…”. That was the question in our case, because every new declared symptom, every new action we took, every new investigation, and every new differential diagnosis failed to reveal the pathology that this child allegedly suffered from. So, we began to wonder about the truthfulness of the declared story.

MSBP represents a severe mental derange. The purpose of these adults is to fabricate diseases for their own children by inventing signs and symptoms or by continuously asking for invasive medical intervention. It is a serious, potentially deadly, form of child abuse. The main alarm signs of this psychiatric pathology are multiple “rendez-vous” with different doctors in different hospitals, and strange symptoms that are never objectified during admission time, and only at home under the supervision of their parent [1,2,3,4,5]. Laboratory investigations performed several times never seem to match with child-described symptoms. The parent might bring to the doctor’s attention several medical documents with numerous diagnoses and investigations that apparently go with the child’s symptoms. In our case, the mother was the only one who “caught” fever episodes and bleeding events, and no medical personnel had ever experienced these symptoms first hand. Child symptoms appeared always at home or night-time during hospitalization and were described by the mother and “documented” with photographs.

Unfortunately, MSBP leads parents to terrible actions, such as adding fresh blood to stool or urine samples, heating the thermometer to mime a fever, giving the child certain medication to induce symptoms (such as insulin to provoke hypoglycemia), starving the child, or worse, contaminating an intravenous line to induce systemic infection [12,13,14,15,18]. Our patient’s mother most likely spread fresh blood all over the child’s shirt during night-time and, claiming the boy was coughing out blood, added fresh blood to urine samples to mimic kidney damage. She managed to make up a systemic vasculitis, the patient underwent repeated, invasive investigations and ended up receiving systemic cortisone therapy. After several hospital admissions, the mother told us she had a medical background—she went to medical school for 3 years.

The DSM-5 defines MHBP with specific criteria, which we elaborated on above in the Introduction: the caregiver falsifies physical or psychological signs or symptoms, or induces injury or disease in the child; the parent presents the victim to others as ill, impaired, or injured; and the deceptive behavior is present in absence of external incentives [2,3,6,8]. In the case presented, the mother described recurrent and inexplicable symptoms, and she was always lurking around for medical attention. She had developed an attachment relationship with the attending physician and other medical personnel. She proudly filled the shoes of the “heroine mother”. She embellished the family’s medical background with every admission and described new symptoms depending on the type of questions the doctor would ask. Every time the discharge moment approached, another aggravating symptom appeared. She was particularly active on social media, where she received validation and occasionally financial support.

MSBP must not be mistaken for another psychiatric pathology such as psychotic or delusional disorder [2,3,6]. The medical team involved in this case excluded various differential diagnoses step by step (Table 1), until a turning point when the elaborate lie became obvious. Because the mother described prolonged fever, alongside a newly emerged symptom every admission, multiple pathologies had to be ruled out: from tuberculosis to congenital immunodeficiency, from PFAPA to idiopathic juvenile arthritis, and from vascular malformations to Goodpasture syndrome. Complex blood, urine, imagistic, and endoscopic investigations had normal results, despite severe complaints.

In this psychiatric condition, a “love triangle” between the mother–child–attending doctor is present. As a doctor, we must always preserve our clear judgment. But, in some cases, when a patient becomes “a friend” of the clinic because of numerous admissions, and has severe undiagnosed pathology, many physicians tend to establish an attachment connection. In such cases, doctors are at risk, because MSBP adults take advantage of the doctor’s human side and use it to their own benefit [7]. That is exactly what the patient’s mother did and it served her well for a period of 4 months. The doctor was dragged, without his will and knowledge, into contributing to invasive investigations and unnecessary therapy. So, there is a talk about ethical and legal issues associated with MSBP.

The mother was unexpectedly calm, patient, and respectful; she was “easy” to like. Despite the child’s continuous aggravating symptoms, she always maintained composure and coolness. The attending physician held her in high regard for a long period of time for how she managed to put up with the difficult situation. Even the medical personnel considered her to a be “heroine” mother, always there for her child, so attentive and kind all the time (despite being herself in a delicate situation—at the time, she allegedly experienced a difficult pregnancy).

A particular clinical circumstance triggered the attending physician’s suspicion. The child was discharged from the Nephrology Department with a very good clinical condition and, in the same evening, he came back to our clinic (where the attending physician was on call) during the night for acute, abundant GI bleeding. An upper endoscopy was immediately performed, but there were no signs of recent or active bleeding. The hemoglobin level was stable all the time, despite all the declared bleeding episodes. This was the turning point—the doctor started to question everything, realized she had never seen the described symptoms (any of the bleeding episodes) first hand, and asked all the medical personnel involved with the case if anybody saw anything with their own eyes, and all the answers were negative. The doctor changed her attitude from empathic to distant, stopped responding to constant phone messages, and, in two days, all complaints vanished (no fever, no bleeding).

The father was never present. The child was always brought by the mother, and they never received any visits during admission time from the father. The mother said that he was away on a military mission. As for the socio-economic status of the family, they seemed to “fit” somewhere in the middle class. The mother was very active on social media, where she often received financial support—she posted about the child’s situation on different social media groups.

In Romania, this syndrome is recognized as a form of child abuse by Decision No. 49 of 19 January 2011: “Munchausen Syndrome by transfer is the artificial creation of a child fake diseases by the parent; disease is induced by administration of drugs for poisoning, or by supporting the existence of symptoms in children who have never been confirmed by the specialists. In both cases, many parents require medical or surgical investigation, abusing the child repeatedly. Any functional sign can be invoked by parents to get painful and intrusive investigations and proceedings for the child” [7,21].

In this case, social services were notified, but with no compelling, hard-proof evidence, no legal measures could be enforced. The legal measures to protect the minor are at this time in the hands of social services and country authorities, but to this day, hard evidence to support this diagnosis is extremely difficult to find. The child was further addressed to the Nephrology Department with a very good outcome (two subsequent evaluations with normal clinical findings and normal blood and urine tests), making the nephrologist conclude that “this is the most surprising and unexplained best evolution of a systemic nephritis ever”.

In Romania, it is extremely difficult to keep track of patient admissions to medical facilities, because we do not have a centralized system. If the patient does not return willingly to the clinic, they are easily lost from follow-ups. We mainly rely on “human connections”, meaning we keep in touch with physicians from other hospitals in order to follow up on patients’ disease courses. It is a “sore problem”, but this is one of the setbacks of our national medical system.

When the attending physician’s and allied health professionals’ attitudes changed from empathic to distant, the mother disappeared from follow-up.

As pediatricians, we have to juggle between child and parents every time. We ourselves must be differently trained and possess unique traits to navigate the muddy waters of adult–child behavior. We need to learn how to get closer but at the same time, how to maintain a safe distance from family’s pain and distress. In particular cases, when a child knocks down your defenses and becomes more than a patient, but a friend. When the doctor opens up and empathizes with his suffering, then it becomes risky, because then, there is room for people like MSBP adults, who take advantage of the physician’s human side and drag him into this deranged road of hurting the child. That is why certain rules must be always followed and certain behaviors must be rejected every time. The medical team must always follow the protocols regarding investigations and treatment. Symptoms that repeatedly do not match with lab or imaging investigation results must trigger suspicion. When there is no explanation of the child’s declared symptoms, this should be a red flag. If any suspicion of adult misconduct towards the child, a psychiatric assessment of the parent should be performed. Physicians must ask for professional help—an evaluation conducted by a psychologist or psychiatrist that states a clear opinion about parent conduct. Pediatrics departments should include doctors of all medical specialties because when a situation like this emerges, we should rely on our fellow colleagues for proper evaluation of parent–child couple; difficult cases would be “put on the table” and discussed with everybody, so there would be no room for error.

## 4. Conclusions

Proper knowledge of MSBP should make clinicians aware and prepared for the day such an intricate case might present itself. Medicine must be based on evidence, signs and symptoms of diseases have to match the expected results of specific investigations. Therapeutic decisions are made based on clinical status and biological or imaging findings. That is why diagnosis and treatment protocols are in place.

A pediatrician’s work largely depends on good communication with the parents. When there is no medical explanation for declared symptoms, one might question the merit of the story. This case sheds a bright light on the difficulty of recognizing MSBP and the severe consequences this disease might have on a defenseless child.

## Figures and Tables

**Table 1:** Different diagnoses excluded over a course of 4 months.

Differential Diagnosis	Described Signs and Symptoms	Exclusion Criteria
Tuberculosis	prolonged fever, night sweats	QUANTIferon repeatedly negative + negative pulmonary X-ray + BK cultures and PCR (−)
PFAPA	prolonged fever + recurrent pharyngeal infections + lymphadenopathy	therapeutic challenge with oral cortisone negative
Idiopathic juvenile arthritis	fever + joint pain + swollen joints	no clinical/time criteria for inflammatory arthritis + no persistent inflammatory syndrome
Congenital immunodeficiency	recurrent admissions for respiratory infections	until 3 years of age no severe recurrent infections + normal leukocyte immunophenotyping
Blood neoplasia	prolonged fever	normal blood count + no atypical cells on blood smear and medullar puncture
Active bleeding	recurrent hemoptysis	no signs of active bleeding on pan-endoscopy
Solid tumors	prolonged fever	complex imaging (CT full body scan, MRI)—normal findings
Goodpasture syndrome and other systemic vasculitis	hemoptysis + massive hematuria	pANCA (**−**) + Anti-GBM antibodies (−) + ANA/DNAdc/RF/CIC (−)

## Data Availability

The original contributions presented in the study are included in the article, further inquiries can be directed to the corresponding author.
